# A Rational Approach for Creating Peptides Mimicking Antibody Binding

**DOI:** 10.1038/s41598-018-37201-6

**Published:** 2019-01-30

**Authors:** Sameer Sachdeva, Hyun Joo, Jerry Tsai, Bhaskara Jasti, Xiaoling Li

**Affiliations:** 1 Department of Pharmaceutics and Medicinal Chemistry, University of the Pacific, Stockton, CA 95211 USA; 2 Department of Chemistry, University of the Pacific, Stockton, CA 95211 USA; 3 Present Address: Amneal Pharmaceuticals, Piscataway, NJ 08854 USA

## Abstract

This study reports a novel method to design peptides that mimic antibody binding. Using the Knob-Socket model for protein-protein interaction, the interaction surface between Cetuximab and EGFR was mapped. EGFR binding peptides were designed based on geometry and the probability of the mapped knob-sockets pairs. Designed peptides were synthesized and then characterized for binding specificity, affinity, cytotoxicity of drug-peptide conjugate and inhibition of phosphorylation. In cell culture studies, designed peptides specifically bind and internalize to EGFR overexpressing cells with three to four-fold higher uptake compared to control cells that do not overexpress EGFR. The designed peptide, Pep11, bound to EGFR with K_D_ of 252 nM. Cytotoxicity of Monomethyl Auristatin E (MMAE)-EGFR-Pep11 peptide-drug conjugate was more than 2,000 fold higher against EGFR overexpressing cell lines A431, MDA MB 468 than control HEK 293 cells which lack EGFR overexpression. MMAE-EGFR-Pep11 conjugate also showed more than 90-fold lower cytotoxicity towards non-EGFR overexpressing HEK 293 cells when compared with cytotoxicity of MMAE itself. In conclusion, a method that can rationally design peptides using knob-socket model is presented. This method was successfully applied to create peptides based on the antigen-antibody interaction to mimic the specificity, affinity and functionality of antibody.

## Introduction

Most cellular processes are mediated by protein-protein interactions. A protein-protein interaction between an antibody and an antigen results in a specific binding of the antibody to a specific antigen with high affinity. The forces involved in the antigen-antibody interface include hydrogen bonds, van der Waals packing, and ionic interactions. Although close steric complementarities are observed in antigen-antibody contact surfaces/interfaces, an induced fit conformation change of the antibody binding site is often observed when bound to antigen. These antigen-antibody interactions cannot be generalized, and their mechanism for specificity is largely an open question. In some cases, only a few large interactions result in strong affinity, while in other cases many weak interactions result in the high affinity of the antibody towards that antigen^1,2^. These findings suggest that the complexity of the antibody-antigen interactions could be predicted by the understanding of the contact interactions between the amino acid residues in antibody-antigen binding interface.

Because of their specificity and affinity, antibodies are commonly used in biomedical research to bind to a specific target protein. In addition to being used as a research reagent, antibodies are also ubiquitous in diagnostic and therapeutic areas. In the past decade, uses of antibody for therapeutic purposes have been rising. As of December 2017, the FDA has approved 73 antibodies as drugs for different therapeutic targets^3^. Recently, a number of antibody-drug conjugates were approved for different cancers, which has led to a new wave of research and product development related to antibodies^4,5^. Since antibodies have high production cost, large molecular size, limited ability to penetrate tumor tissues and side effects such as immunogenicity^6^, the development of antibody mimics and alternatives have been an attractive area for specific binding molecules. Antibody alternatives or artificial antibody mimics include the fragment antigen binding region (Fab)^7,8^, variable fragment (Fv) and single chain variable fragment (ScFv)^7,9,10^, nanobodies^11,12^, synthetic antibodies^13^, aptamers^14^, and small peptides.

Peptides that can bind to specific targets have been obtained by structure free screening or structure based design. Structure free techniques include phage display, RNA display and other screening methods that are time consuming and trial-and-error in nature with uncertain outcomes. In a study by Diehnelt *et al*., a new peptide based synthetic antibody (synbody) was developed and screened against an array of 8000 human proteins to identify the binding proteins for this synbody. The binding of synbody in this study did not begin with a specific target. Instead, the target was determined after the screening. The synbody was found to bind to AKT1^15^. Williams *et al*. identified another synbody by screening an array of 4000 different 12 residue peptides against GAL80. In this study, a massive number of peptides were used to search for one or more peptides that bind to a specific target. These peptides were conjugated through a DNA scaffold to make the synbody^16^. Song *et al*. identified a peptide ligand against EGFR using a computer aided design approach and a large virtual peptide library screened against the crystal structure in silico^17^. In a study by Honarvar *et al*., a HER2 specific peptide A9 with nanomolar affinity was derived from transtuzumab-Fab portion and was used as a ^111^In radio labeled imaging probe for HER2 positive tumor tissue^18^. Although these peptides bind with high affinities (K_D_ in nanomolar range) the studies involve tedious and time consuming *in vitro* or in silico screening process to identify the peptides against a target. Most importantly, the target binding peptides in these studies were obtained by a random search instead of rational design.

In contrast, structure based peptide design utilizes the crystallographic structural information from the interface of a protein-protein interaction to direct the design of a peptide’s binding specificity. This approach often uses computational modeling and docking studies as tools in peptide design. A common drawback for various computational methods has been a lack of meaningful description of packing interactions between the contacting amino acid residues. To address this challenge, current approaches apply multiple random iterations to determine/sample the amino acid packing in peptide-protein interactions. Moreover, the binding constant of the designed molecules from these models remains in micromolar range affinities^19^.

**Figure 1:**
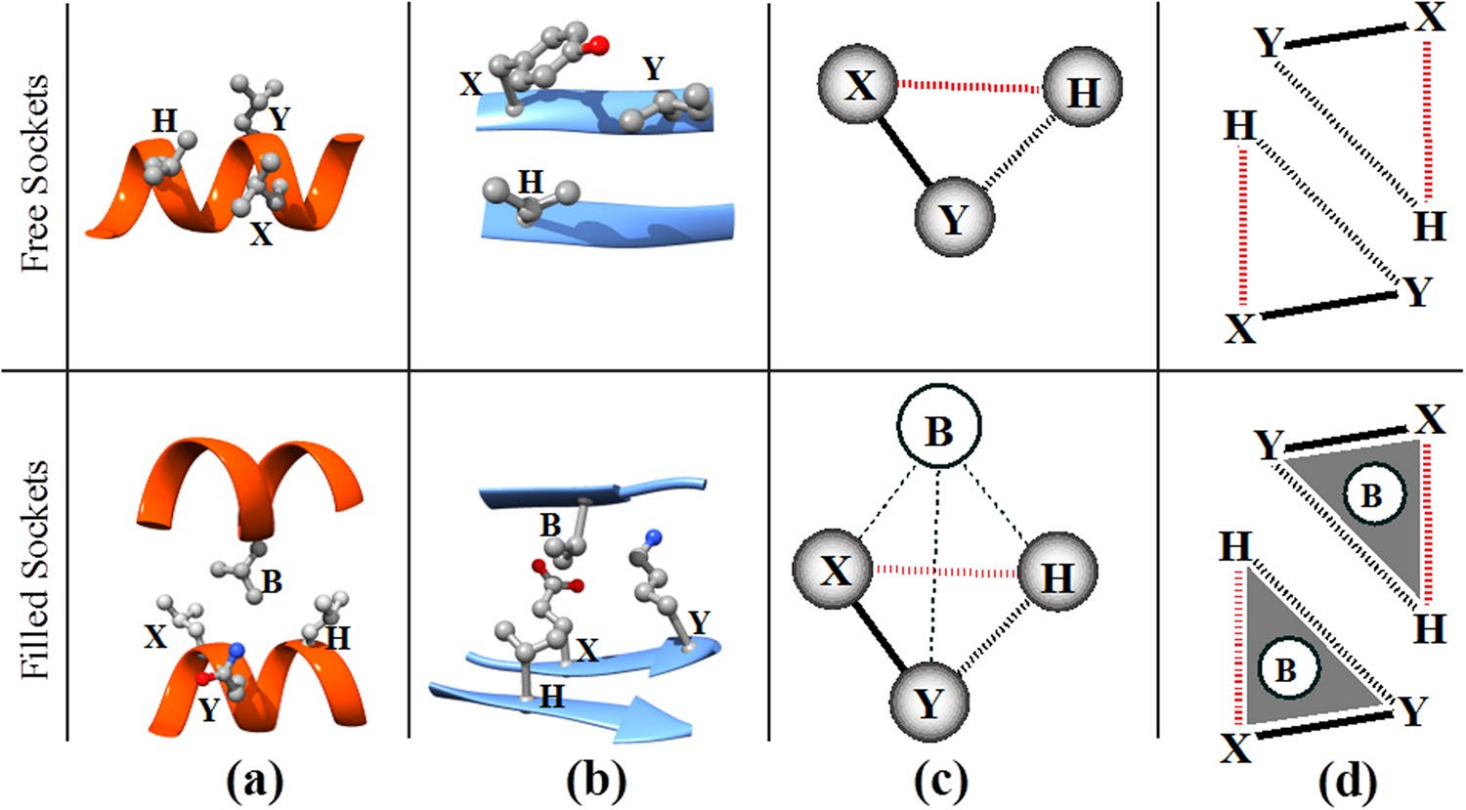
Knob-Socket motifs in α-helix and β-sheet. Free sockets are shown in the top panels and filled sockets with a packing knob are shown in the bottom panel. Ribbon representation with ball-and-stick representations of amino acid residues forming a free socket and a knob-socket motif in α-helix (**a**), and in β-sheet (**b**), X, Y, and H represent the amino acid residues in contact forming a socket, XY:H, and B represents a knob, a residue packing into a socket. The 3 dimensional configurations of socket and knob-socket motifs are shown in (**c**) and the 2 dimensional representations of socket and knob-socket are shown in (**d**).

The understanding of protein-protein interactions could benefit from a more intuitive protein packing model. The knob-socket model^20–23^ simplifies packing into easily understood patterns of a four-residue motif, where a single residue knob from one secondary structural element packs into a socket formed by three residues in another secondary structure as shown in Fig. 1. The knob-socket motif has been proven insightful in describing protein secondary packing structure not only in α-helices^20^ and β-sheets^21^ but also between mixed secondary structures^22,23^. Knob-socket model also describes the three dimensional complexity of packing and gives information about the preference of amino acid knobs packing into respective sockets. Therefore, this knob-socket code can be applied in the rational design of a peptide that can bind to a specific site of a protein by mimicking the packing mode of protein-protein interaction.

Therefore, the aim of this study is to demonstrate effectiveness of a knob-socket analysis and develop a novel method in overcoming the uncertainty of current screening methods for finding peptides that can bind to a defined target. In an analysis of a co-crystal structure, the knob-socket model allows a clear definition of the contribution of the residues involved in the interaction between an antibody and antigen. Epidermal growth factor receptor (EGFR) is used as a model target in this study. The interaction interface is mapped into contributing residues as knobs from the antibody and socket surfaces on the EGFR antigen. From this analysis, peptides could be designed in a straightforward manner that mimics the antibody binding. Essentially, a set of rationally designed peptides are produced based on the knob packing preferences of the sockets on the EGFR surface calculated from the protein structure. By considering the geometry and the distance between the sockets on the EGFR epitope, the individual knob residues were connected by bridging amino acids to complete a linear peptide. A set of peptides were tested computationally to assess their potential binding. A selection of the initial set of peptides were synthesized and characterized experimentally for their binding affinity specificity and functionality to EGFR.

## Results

### Design of novel peptides based on knob and socket model

**Figure 2:**
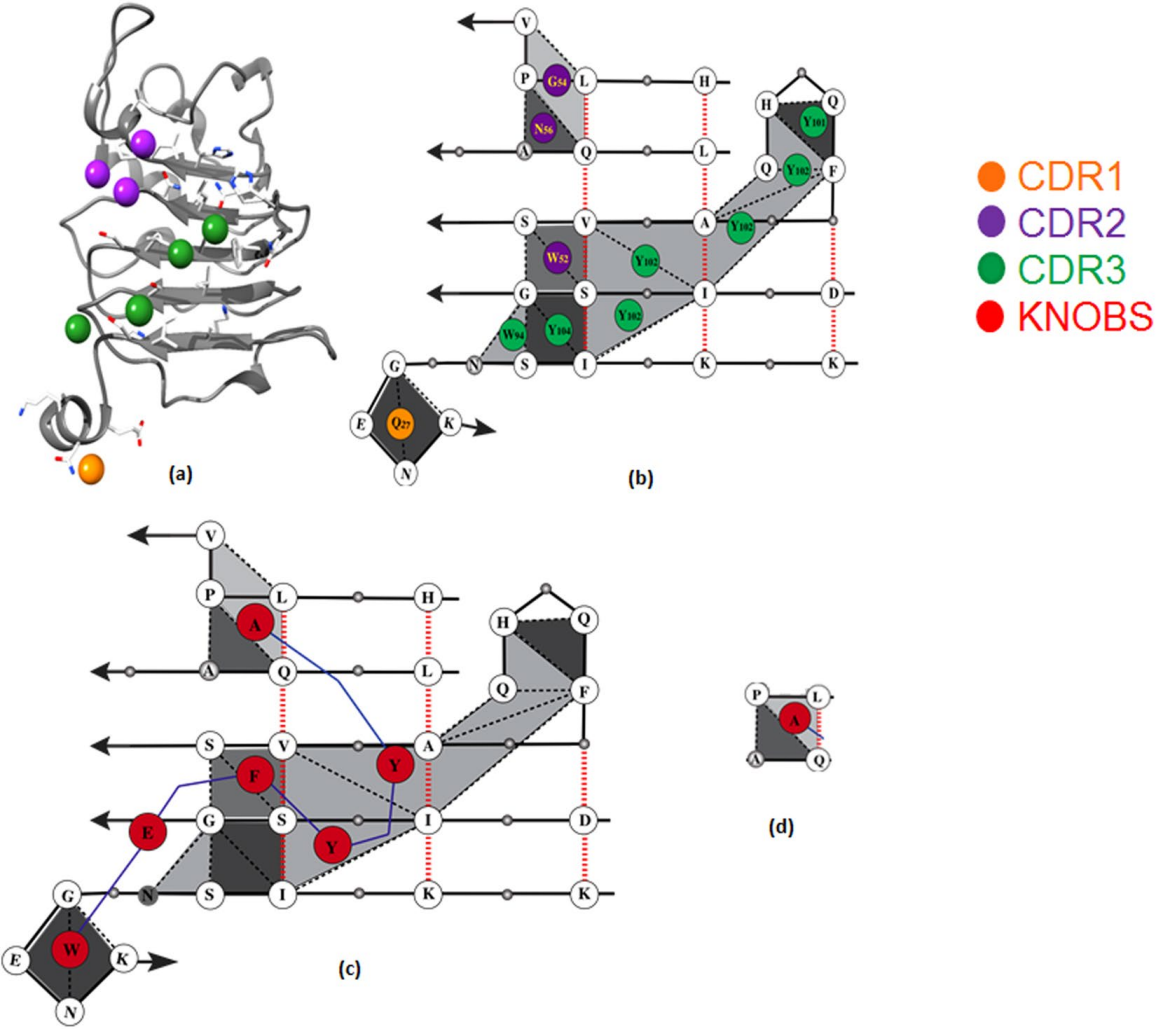
Ribbon diagram of the Cetuximab-EGFR interface (**a**) and schematic of the interaction map diagram. (**b**) Schematic of peptide design representing different Knobs and Sockets. (**c**) AQ:P and LP:Q in white circles act as sockets and Alanine (A) in red circle acts as a Knob (**d**).

The knob-socket data of the crystallographic three dimensional structure of Cetuximab and EGFR complex (PDB id: 1YY9)^24^ were calculated. Using the knob-socket model (Fig. 1), the three-dimensional packing interface of Cetuximab and EGFR (PDB id: 1YY9)^24^ was represented into two-dimensional packing surface topology map where knobs from Cetuximab are projected onto sockets on the EGFR binding surface as shown in Fig. 2. The set of three residue sockets on EGFR that interact with single residue knobs from Cetuximab clearly defines the epitope surface. This interaction surface is shown as grey triangles, while free sockets without any interaction are shown in white. The amino acid residues serving as knobs from Cetuximab have been labeled with orange, purple, and green, to indicate the location of these knobs from the different CDR regions and non CDR region (red) as shown in Fig. 2(b,c). Figure 2(c) shows a schematic knob-socket map for peptide design that replaces the amino acids with propensity for EGFR epitope sockets with higher propensity residues. The propensities to identify the best knob residues to pack in each socket were obtained in the previous studies^20–23^.

An initial set of 24 peptides resulted from this knob-socket based design approach were computationally analyzed using docking methods. Four peptides (Pep6, Pep11, Pep22, and Pep24) as shown in Table 1 were advanced to further study based on the binding energy and preserved knob-socket interactions. As a negative control, Pep25 was designed with the same amino acid composition as Pep11, but a scrambled sequence.

**Table 1:** Peptide sequence with docking results.

Peptide	Selected Peptides	Binding Energy (kcal/mol)	Total Interactions	Preserved Interactions
Pep6	WSGENGPGYFDYEA	−32.47	13	4
Pep11	WSGENGPGFYDYEA	−40.43	17	8
Pep22	WSGENGPGTFYDYEA	−35.34	14	6
Pep24	AEYDFYGPGNEGSW	−40.31	13	4
Pep25	SGEWAYDGYEPNFG	−23.69	7	2

### Binding specificity: Cellular uptake study

**Figure 3:**
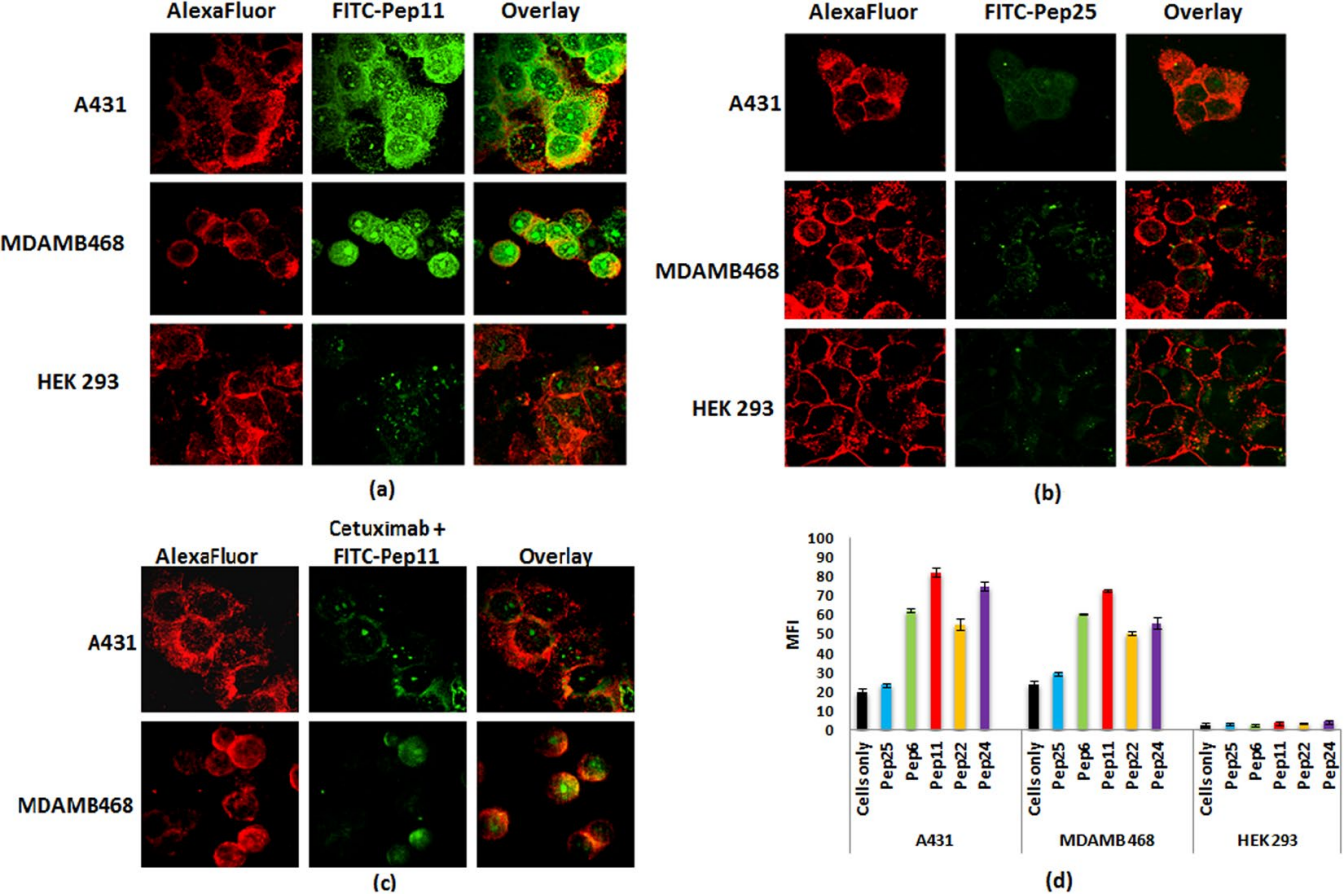
Binding specificity results: Confocal images of Alexa Fluor column represents the cell treated with only Alexa Fluor 594 membrane dye, only FITC-Pep11/Pep25 and overlay column represent the merge of the two images. (**a**) displays the cells treated with FITC conjugated Pep11, (**b**) displays the cells treated with FITC conjugated control Pep25, (**c**) displays the cells pretreated with Cetuximab for 30 mins and then treated with FITC conjugated Pep11 and (**d**) displays the flow cytometry results displaying mean fluorescent intensity for different treatments and cell lines.

In the binding specificity study, confocal microscopic images showed that all designed peptides specifically bound to A431 and MDA MB 468 cells that overexpress EGFR and internalized into the cells. The designed peptides showed negligible binding to the HEK 293 cell line which does not overexpresses EGFR as shown in Fig. 3(a,b). These images also showed significantly higher uptake of peptides in A431, MDA MB 468 cell lines as compared to HEK 293. Figure 3(c) showed A431 cells and MDA MB 468 cells displayed a lower uptake of Pep11, when the cells were pretreated with Cetuximab for 30 minutes as compared to the cells when they were not pretreated with Cetuximab. A negative control, Pep25 which has a scrambled sequence of Pep11, shows negligible binding to all cell lines.

Flow cytometry results showed the cellular uptake of peptides was 3 to 4 folds higher in the EGFR overexpressing A431 and MDA MB 468 cells compared to control peptide (Pep25) as shown in Fig. 3(d). Pep11 showed the maximum uptake with 1.5 fold higher fluorescence as compare to other peptides, while negligible uptake of these peptides was observed in non-EGFR overexpressing HEK 293 cell line. Control peptide (Pep25) with scrambled sequence of Pep11 showed negligible uptake to all cell lines. A431 and MDA MB 468 cells showed higher fluorescence intensity as compared to HEK 293 cells. This could be attributed to the higher auto fluorescence emitted by these cell lines which was clearly observed in the florescence intensity of cells only samples.

### Affinity: Binding constants analysis by surface plasmon resonance (SPR)

**Figure 4:**
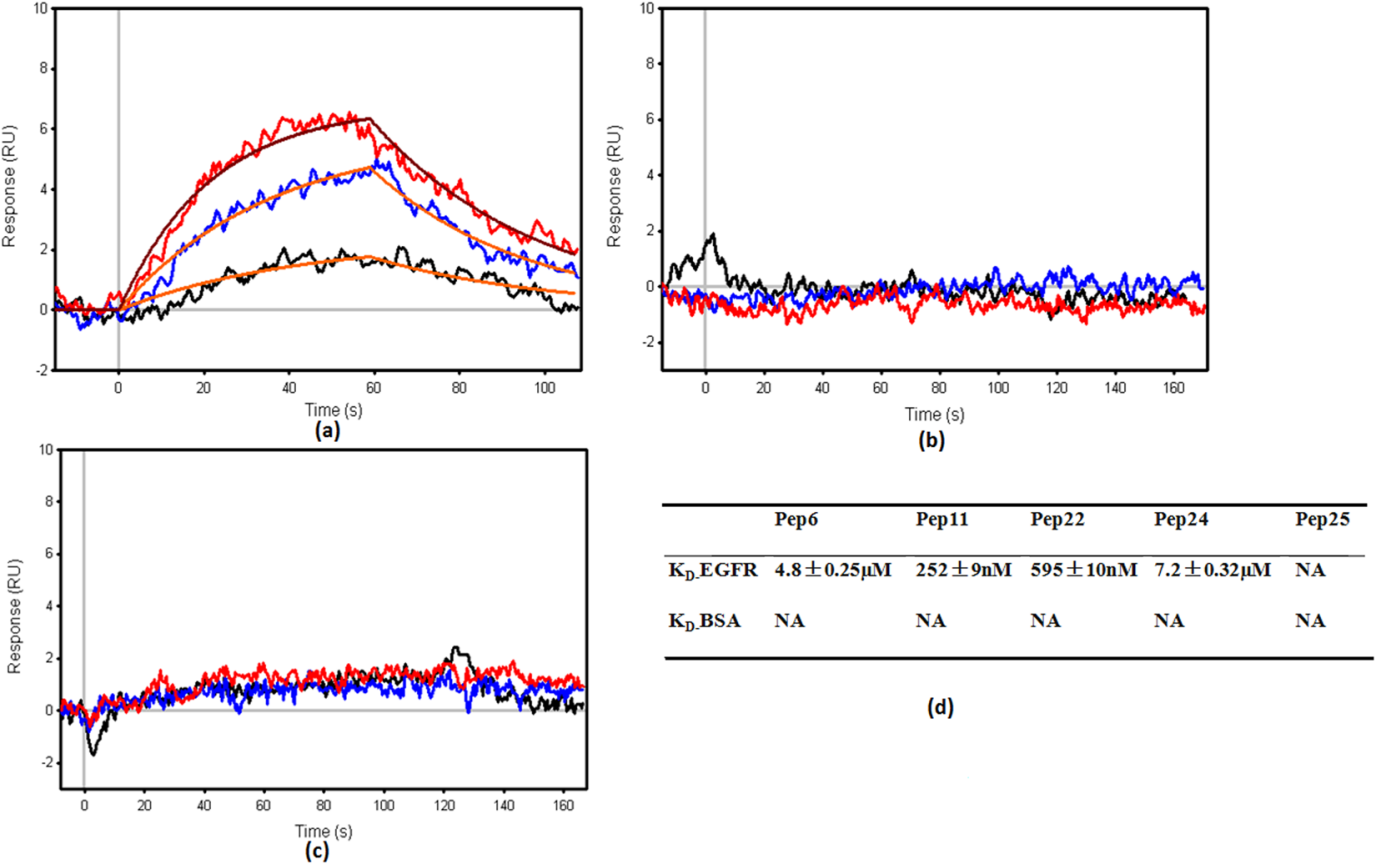
SPR sensograms for Pep11 on EGFR as immobilized surface, (**a**) SPR sensograms for Pep11 on BSA as immobilized surface, (**b**) sensograms for Pep25 on EGFR as immobilized surface. (**c**) Binding affinity (K_D_) values for selected peptides (**d**).

Binding affinity constants determined by using SPR for the designed peptides ranged from 252 nM − 4.8 µM. Pep11 had the best binding affinity with K_D_ of 252 nM against soluble EGFR protein as shown in Fig. 4(a). Fastest k_on_ was also observed with Pep11. Affinity constant of Cetuximab was 5.2 nM^25^, which is about a 50 fold difference than the K_D_ of Pep11. Affinity constant of EGF was reported as 130 nM^24^. A 2 fold difference is observed between K_D_ of EGF and Pep11.

Pep22 with extra threonine residue than Pep11, and Pep 6 with tyrosine and phenylalanine residues switched from Pep11 showed a lower biding with K_D_ of 595 nM and 4.8 µM respectively as compared to Pep11. Pep24 with a reversed sequence of Pep11 showed least binding with K_D_ of 7.24.8 µM. A 30 fold difference in K_D_ values was observed among all tested peptides. Pep11 did not show binding to bovine serum albumin (BSA) as seen in Fig. 4(b). The control peptides did not show binding to the EGFR and no increase in response was observed with increase in concentration of the control peptide as shown in Fig. 4(c).

### Functionality: EGFR phosphorylation studies of peptides

**Figure 5:**
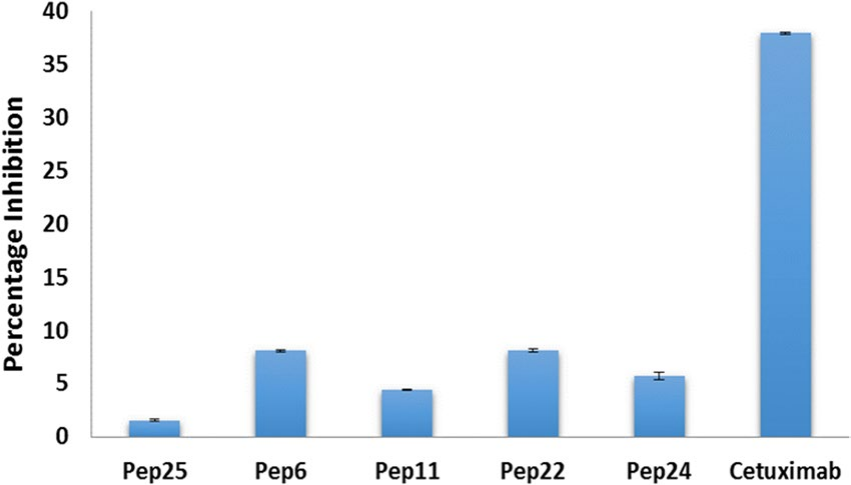
Percentage inhibition of phosphorylation by different peptides and Cetuximab after stimulation by 50 ng/ml EGF.

Like Cetuximab, the designed peptides showed a decrease in EGFR phosphorylation (Fig. 5). Percent inhibitions of EGFR phosphorylation by the designed peptides were found to be in the range of 4.4–8.1%, when stimulated by EGF. Cetuximab inhibited the EGFR phosphorylation by 38% as depicted by Fig. 5. A 4.5 fold difference was observed between Cetuximab and peptides in this functionality study.

### *In vitro* cytotoxicity of peptide-drug conjugate for EGFR targeted delivery

**Figure 6:**
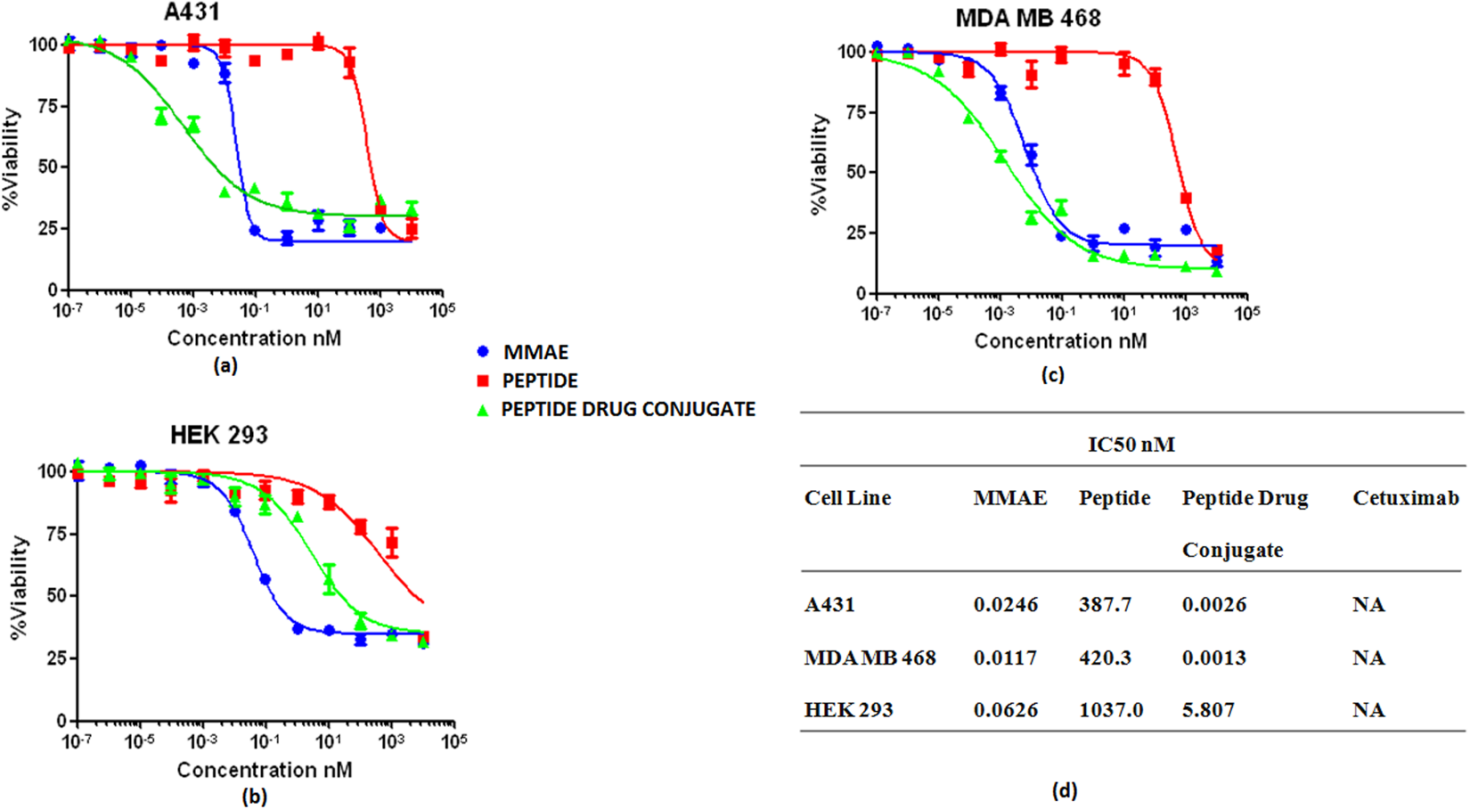
Cytotoxicity study: Percent Viability vs Concentration plots MMAE, Pep11 and MMAE-Pep11 on A431 Cell line, (**a**) MDA MB 468 cell line, (**b**) HEK 293 Cell line (**c**) and IC_50_ value of MMAE, Pep11, MMAE-Pep11 and Cetuximab across different cell lines (**d**).

A conjugate of monomethyl auristatin E (MMAE) and Pep11 (MMAE-Pep11) had IC_50_ of 0.0026 nM, 0.0013 nM and 5.807 nM for A431, MDA MB 468 and HEK 293 cells, respectively as shown in Fig. 6(d). The cytotoxicity of the conjugate towards EGFR overexpressing cell line is more than 2000 fold potent when compared to control HEK 293 cells. The IC_50_ of MMAE alone was found to be 0.0246 nM, 0.0117 nM and 0.0626 nM for A431, MDA MB 468 and HEK 293 cells, respectively. The MMAE-Pep11 conjugate showed ~10-fold higher cytotoxicity than the drug MMAE itself against EGFR overexpressed cells A431 and MDA MB 468. On the other hand, MMAE-Pep11 conjugate showed more than 90-fold times lower cytotoxicity towards non-EGFR overexpressing HEK 293 cells when compared with cytotoxicity of MMAE itself as shown in Fig. 6.

## Discussion

The current practice for obtaining a peptide that would bind to a specific target is by trial and error approaches, such as massive screening by phage and RNA display. These methods are labor intensive and resource-demanding. For the phage display method, a library of peptides with large number of peptides with different amino acid combinations is usually used in searching for a peptide that binds to a target by screening. Uncertainty of outcomes is the major drawback of this type of methods.

Rational design of peptides based on the specific residue interactions between antibody and antigen is a more straight-forward and direct approach to identifying peptides that bind with a specific target. In this study, the knob-socket model is used to map the specific interactions between antibody knobs that pack into the sockets on interaction surface of an antigen. Peptides are designed based on the knobs that fill or bind to a specific region on the antigen surface. In the design step, new knob residues were selected from 4,753,641 knob-socket packing cliques^20–23^ to construct new peptides that can bind specifically to the intended target. This approach allows the creation of desired peptide by rational design instead of by a random sampling method.

The effectiveness of using this design approach to create target specific binding peptides is demonstrated by the results of *in vitro* binding specificity, affinity, and functional studies. Higher fluorescence intensity of designed peptides in A431 and MDA MB 468 cells than HEK 293 cells in confocal microscopic and flow cytometric studies displayed their binding specificity towards EGFR.

Negligible binding/uptake to the EGFR overexpressing cell lines of the scrambled sequence peptide Pep25 that has the same amino acid composition as Pep11 and Pep6 demonstrates the necessity of specific sequence of amino acids by this rational design approach. Designed peptides also specifically bound to the soluble recombinant EGFR protein in the SPR binding study with affinity up to nanomole range, while the scrambled sequence peptide Pep25 did not bind to soluble recombinant EGFR protein in SPR study as shown in Fig. 4. Higher binding of designed peptide Pep11 and non-binding of the scrambled Pep25 indicate that the sequence, the order of amino acid residues as identified by the knob-socket mapping is required for specific binding. This result also led us to believe that Pep11 is most likely bind to the intended interaction surface. These results demonstrate the selectivity and specificity of the designed peptides towards EGFR. Lower fluorescence intensity in confocal microscopic studies for A431 and MDA MB 468 cells, when EGFR was blocked by pre-incubating the cells with Cetuximab, showed that the designed peptides competed for the same binding site. Therefore, the inhibition or decrease in the binding of peptides or antibody mimics after Cetuximab treatment corroborated that the binding of antibody mimic Pep11 to the same EGFR domain III site as that of Cetuximab as intended by design. The results from Cetuximab pretreatment study provide a strong support for our rational design approach.

Affinity studies provided information on how fast and strong the antibody/peptides bind to the target protein. The small size of the designed peptides resulted in the faster binding association k_on,_ which could be highly desired for quick binding to the target in drug delivery. The results of SPR study show that the peptides have strong potential in targeting EGFR with affinities in the nanomolar range.

The binding affinity is sensitive to the sequence of peptide. A change in the sequence of the designed peptide resulted in different binding affinity constants. For example, Pep11 showed the best binding in this study due to predicted packing of the knobs phenylalanine and tyrosine residues for sockets of amino acids VS:S and SI:I on EGFR, respectively. When Tyr and Phe were swapped in Pep6 (See Table 1 for the sequences of peptides), the binding constant decreased by 19 fold. The designed knob and socket binding pairs preserved in the docking study was used as an indicator to evaluate the tendency of the designed peptide binding to the intended target. Better binding of Pep11 also reflected in the binding energies in the docking studies when compared to Pep6. Pep11 has the lowest calculated binding energy and maximum preserved interactions among rest of the designed peptides. Pep6 had a higher binding energy and fewer preserved interactions. The overall high affinity of Pep11 could be attributed to the design of peptide using high probable knobs to fit the sockets on the binding site. These results provide the foundation and also demonstrate the sensitivity for the rational design of peptides using the knob-socket model.

Cetuximab binds to the domain III of extracellular region of EGFR and blocks the binding of EGF, hence inhibits the dimerization and phosphorylation. This blocking of EGF binding and inhibition of EGFR phosphorylation results in the antitumor activity of Cetuximab. As shown in Fig. 5, a decrease in the phosphorylation caused by the different peptides exhibited the similar functionality of Cetuximab. However, the percent inhibition of designed peptides was about 4.5 folds less as compared to the Cetuximab. Although the designed peptides can bind to the similar site as Cetuximab, they can only exhibit partial inhibition function. Since designed peptides were 70–80 folds smaller than Cetuximab in size, the lower inhibition of phosphorylation may be attributed to its smaller size that resulted in inefficient inhibition of the EGFR conformational change. Foy *et al*. had observed similar results when studying a peptide with molecular weight of 3500 Da generated directly from contact sites between EGF and EGFR. The percentage of EGFR phosphorylation inhibition by peptides in the study was 2–3 folds less as compared to Cetuximab’s inhibition which was around 50%^26^. Given the molecular weight of peptides (1600–1700 Da) in this study, the reduction of phosphorylation functionality by the designed peptides was reasonable and it did demonstrate the capability of designed peptides to bind to the intended target site in EGFR. Therefore, the partial functionality can also be seen as an indirect evidence for the specificity and affinity of these designed peptides. Although the functionality of peptides is not as potent as antibody, the binding specificity and reasonable affinity of these peptides can be used for other applications to mimic antibody with needs in binding specificity, such as serving as targeting moiety for targeted drug delivery.

Antibodies have been shown to bind with a very high specificity and affinity to their target antigen, but most of these antibodies display insufficient cytotoxicity^27^. As a result, antibodies are generally used in conjunction with chemotherapy or conjugated with an anti-cancer agent for targeted delivery in cancer therapy. In an antibody-drug conjugate, the antibody is primarily utilized as a specific binding moiety. Antibodies have both high specificity and affinity, but the specificity is the characteristic desired for targeted delivery. Therefore, a much smaller molecule with the same or similar specificity that binds faster has merit in targeted drug delivery. In addition to the binding specificity, ease of synthesis, better tolerance towards pH and temperature, and flexibility towards chemical modification are other factor to be considered in selecting a specific binding moiety for targeted delivery. Due to the low molecular weight and small size, peptides have higher tumor penetration or uptake than antibodies. Moreover, peptides are less toxic and have low immunogenic potential. These advantages make the specific binding peptides an ideal candidate to be used as imaging probes and targeting agent.

In this study, the peptides designed based on knob-socket interactions in the antibody-EGFR co-crystal structure were used to create the targeting moiety of peptide-drug conjugate. A highly potent and non-specific anti-cancer drug, MMAE, was conjugated with peptide that mimics Cetuximab specificity for EGFR. *In vitro* cytotoxicity studies with the MMAE-Pep11 conjugate demonstrated that this peptide-drug conjugate can selectively kill EGFR overexpressing cancer cells while having lower toxicity towards non EGFR expressing normal cells. The increase in the difference of cytotoxicity of MMAE-Pep11 and MMAE to cancer cells and normal cells showed the selectivity of peptide-drug conjugate and form the basis for targeting the delivery of MMAE to EGFR expressing cancer cells. The sensitivity of peptide-drug conjugate to the EGFR overexpress cell line further supports the binding specificity results obtained in the cellular uptake, SPR, microscopy and flow cytometry studies. This *in vitro* cytotoxicity studies support the feasibility of using a rational design approach to create antibody mimic for targeted drug-delivery.

## Conclusion

In this study, a novel method for the rational design of peptides to mimic antibody binding based on the knob-socket model has been established to circumvent the uncertainty of current trial and error methods in searching for target specific binding peptides. Rationally designed peptides satisfy all the three important characteristics of antibody binding i.e. specificity, affinity and functionality. The antibody mimic peptides were successfully utilized as a targeting moiety via a peptide drug conjugate approach for targeted delivery of an anticancer agent to EGFR overexpressing cells.

## Methods

### Design of peptides by using knob-socket model

The crystallographic structure of Cetuximab-EGFR (PDB id: 1YY9)^24^ was used to define the interactions between Cetuximab-EGFR. Crystal structure of antigen-antibody complex was visualized using UCSF Chimera program package^28^. A Knob-Socket analysis defined all the sockets formed on the surface of EGFR and all knobs formed by Cetuximab in the following procedure outline in previous work^20–23^. An in-house program was used to precisely define a set of residues that all contact each other and classify them based on contact order^29^. From the coordinates of the co-crystal structure, atomic contacts were calculated from Voronoi polyhedra^30^, and residue-residue contacts were built up from the atomic contact information. For the residue contacts, a graph was constructed from the resulting Delaunay tessellation of residue interactions^31^. Packing cliques were defined from this graph by finding sets of residues that all contacted each other and these packing cliques were classified based on contact order. This classification produces the set of knob-sockets in the Cetuximab-EGFR structure shown in Fig. 2. For this study, only sockets and knobs formed in the binding interface between Cetuximab-EGFR were used for peptide design. In particular, knobs came from the Cetuximab antibody that bound the sockets forming the antigen binding surface. These are mapped out in Fig. 2.

As shown in Fig. 2, peptide antibody mimics were then designed by linking the amino acid knob residues mapped from the Cetuximab antibody that pack into the EGFR surface sockets. The three residue sockets are then used to query the knob-socket packing propensities to identify the best knob residues to pack in each socket^20–23^. Different combinations of amino acids from the list of preferred knobs are used to make peptide sequences. To connect all the knob residues into a single peptide requires residues to bridge the physical distance between them. Amino acids are inserted to fill the gaps based on the distance between the residues. An average Cα-Cα distance of 3.8 Å was used, and the designed peptide is assumed to take on a random coil structure. Different combinations of amino acids from the list of preferable knobs are used to make multiple peptide sequences and 24 peptides were designed according to knob-socket model.

### Selection of peptides according to docking energy analysis

Computational studies were carried out using Molecular Operating Environment (MOE) program package^32^. The stochastic conformational searches were carried out for all the designed peptides, and the minimization was carried out to prepare the peptides for the docking studies in MOE. The receptor protein, EGFR was prepared for docking by removing bound antibody, organic molecules, and water molecules from the crystal structure (PDB id: 1YY9). For the docking, the entire extracellular region of EGFR was defined as the possible docking pocket on EGFR. The conformations of antibody mimic-EGFR complex with lowest docking energy were then analyzed in terms of binding energy, total number of interactions, preserved interactions between antibody mimics and EGFR by MOE. Peptides showing relatively strong binding energies and higher number of preserved interactions with EGFR which are the same as those of Cetuximab with EGFR complex were then selected as shown in Table 1.

### Synthesis and characterization of peptides and FITC conjugations

Peptides were synthesized by solid phase synthesis using Fmoc chemistry on the Wang resin and the coupling of amino acids was performed with 1-hydroxy-benzotriazole (HOBt) and diisopropyl-carbodiimide (DIC). Wang resin preloaded with the C-terminal end amino acid of different sequences were swelled in DMF first, the amino group of that amino acid was deprotected using 20% piperidine in DMF for 30 minutes. After washing with DMF and DCM for three times, the Fmoc protected incoming amino acids was added with 3 equivalents of DIC and HOBT for 3 hours. The Kaiser test was used to confirm the completion of the coupling of that amino acid by observing the color of resin. The deprotection and coupling procedures were repeated until the last amino acid at end of N-terminal was added on to resin.

At last, the resin was washed and the peptide was cleaved from the resin by adding the cleavage cocktail of TFA:TIS:H_2_O (95:2.5:2.5) and reacting for 3 hrs. The eluted solution was added dropwise into ice-cold diethyl ether to form precipitation of peptides. The precipitated peptide was collected by centrifuge and washed three times with ice-cold diethyl ether to remove any residual scavengers. The peptides were all dissolved in distilled water and lyophilized.

Fluorescein isothiocyanate (FITC) was conjugated to the peptides by using a 6-aminohexanoic acid linker (Ahx) at the N-terminus of the last amino acid. The lyophilized peptides (unlabeled and labeled) were dissolved in water with acetonitrile and purified by using Reversed-phase HPLC on a C18 column with a linear gradient of solvent A (0.1% TFA⁄H_2_O) and solvent B (acetonitrile). The peptides were characterized using ESI/(MALDI) mass spectrometry.

### Binding specificity: Cellular uptake study

#### Confocal microscopic studies

Confocal microscopic studies were performed on EGFR positive A431 (epithelial carcinoma cell line) and MDA MB 468 (human breast cancer cell line) and HEK 293 (human embryonic kidney cell line) cell as control. A431, MDA MB 468 and HEK 293 cells were cultured in Dulbecco’s modified Eagle’s medium (DMEM) with 10% fetal bovine serum (FBS), 1% glutamine and 1% penicillin-streptomycin.

All the cells were grown in T75 flasks at 37 °C and 5% CO_2_. Upon reaching 80% confluency, the cells were seeded on to coverslips placed inside 6-well culture plates at a density of 80,000 cells/well. After 24 hours, cells were washed with HBSS and treated with designed peptides and control peptide (10 μM).

After incubating the cells with designed peptides for 15 minutes at 37 °C, the medium was removed and the cells were washed with HBSS. In case of competitive binding study, EGFR in A431 and MDA MB 468 cells were blocked by pre-incubating the cells with Cetuximab for 30 minutes. Finally after washing the cells with HBSS these A431 and MDA MB 468 cells were treated with FITC labeled designed peptides for 15 minutes.

The cells were then treated with a 2.5 µg/mL solution of Alexa Fluor 594 wheat germ agglutinin in HBSS for 10 minutes to stain the cell plasma membrane. The dye solution was removed and cells were and then fixed using 4% paraformaldehyde solution prepared in HBSS. The cells were washed with HBSS followed by a final wash of distilled water. A 10 μL drop of the mounting medium (slow fade gold) was placed on the microscopic slides and the coverslips with the fixed cells were placed on the slides. The coverslips were then sealed on all four sides.

The slides were imaged on a confocal laser scanning microscope (Leica Microsystems GmbH, Germany) using 65X magnification and oil. The FITC fluorescence was visualized using 491 nm and the Alexa Fluor 594 fluorescence was visualized using 561 nm wavelength filter. Images were taken at 65X under oil immersion and analyzed with MetaMorph (Molecular Devices, LLC.) software.

### Binding specificity: Cellular uptake study

#### Flow cytometric studies

Flow cytometric studies were carried out to quantify the fluorescence intensity of cells after incubated with FITC labeled mimics. A431, MDA MB 468, and HEK 293 cells were seeded in 6 well culture plates with density of 3 × 10^5^ cells per well for 24 hours and then incubated with FITC labeled peptides at concentration of 10 µM in HBSS at 37 °C for 15 minutes. After washing with HBSS twice, cells remained on the plates were trypsinized with Trypsin-EDTA and centrifuged at 1500 rpm for 5 min. Supernatant were removed and cell pellets were resuspended in 1 ml of HBSS. The suspension was transferred to BD FACSCalibur flow cytometer (BD Biosciences, CA, USA) and cell fluorescence was measured with high dynamic range photomultipliers with 530 nm filter. Quantitative changes of fluorescence in different cells samples were assessed by mean fluorescence intensity (MFI) and FACS data were analyzed using BD CellQuest Pro software (BD Biosciences, CA, USA).

### Affinity: Surface plasmon resonance study

Surface plasmon resonance (SPR) studies were conducted using the Dual Channel SPR Spectrometer SPR7000DC (Reichert Technologies, New York, USA) to determine the binding affinity of peptides to EGFR protein. All experiments were carried out at 25 °C. Phosphate buffer saline (PBS) with 0.01% Tween 20 (pH7.4) was filtered and thoroughly degassed before use. EGFR (20 µg/mL) recombinant human protein and bovine serum albumin (BSA 20 µg/mL) were covalently attached to the left channel of 500,000 Da carboxymethylated dextran sensor chips using 1-ethyl-3-(3-dimethylaminopropyl)-carbodiimide (EDC) and N-hydroxysuccinimide (NHS) chemistry. The chips were then blocked by passing ethanolamine for 8 minutes at a rate of 10 µL/min.

All peptides were injected as soluble analytes in PBS with 0.01% Tween 20 at different concentrations with an injection speed at 15 µL/min. A control peptide Pep25 was also injected in the SPR studies. The bound surface was regenerated by sequential injections of 10 mM glycine in PBS (pH 2.5).

The sensorgrams from SPR were globally treated using Scrubber 2 software (BioLogic Software Pty Ltd, Australia), and kinetic constants k_on_ and k_off_ were determined by global fitting analyses of the titration curves using the 1:1 Lagmurian interaction model considering the mass transport. The equilibrium dissociation constant (K_D_) was calculated from the k_off_/k_on_ ratio.

### Functionality: EGFR phosphorylation studies of peptides

Cell based ELISA was performed to determine the inhibition of EGFR phosphorylation using Abcam’s human EGFR ELISA kit. Briefly, 2 × 10^4^ A431cells were seeded in 96 well plates in DMEM medium supplemented with 10% FBS for 24 hours. The medium was then discarded and was replaced with serum free DMEM medium overnight. The cells were then treated with different peptides and Cetuximab 10 µM in serum free medium for 45 minutes at 37 °C. After that, cells were stimulated by EGF 50 ng/mL for 20 minutes at 37 °C. After washing the cells, fixing solution was added for 20 minutes at room temperature.

Quenching buffer was then added and plate was incubated for 20 minutes at room temperature followed by the addition of blocking solution for 1 hour at 37 °C. Primary mouse anti EGFR and antiphospho-EGFR antibodies were added and cells were incubated overnight at 4 °C. Cells were then treated with anti-mouse HRP secondary antibody for 1 hour at room temperature. Finally, after washing TMB solution was added for 30 min at room temperature followed by the addition of stop solution. The absorbance of the resulting solution was then immediately read at 450 nm. Inhibition of phosphorylation was calculated using the following equation 1:

1 $$ \% \,{\rm{Inhibition}}=\frac{(OD\,Untreated)-(OD\,Treated)}{(OD\,Untreated)}\ast 100$$

### EGFR-PEP11 –MMAE drug conjugate

#### Synthesis of peptide-drug conjugate

Step1. Synthesis of Auristatin E: Monomethyl auristatin E was dissolved in dioxane:water (1:1), and 0.42 mmol of 37% aqueous formaldehyde and 0.13 mmol of sodium cyanobohydride were added to drug solution. The pH was adjusted to 6–7 with 0.1 N HCl and the mixture was heated at 100 °C for 2 hours. The reaction mixture was then poured into a saturated sodium bicarbonate aqueous solution and ethyl acetate. The ethyl acetate layer was separated and dried by adding sodium sulphate. A white crystalline powder was obtained.

Step2. Synthesis of 5-benzoylpentanoic ester of Auristatin E: Auristatin E (0.07 mmol) was dissolved in anhydrous methylene chloride, followed by the addition of DCC (0.14 mmol) and DMAP. 5-benzoylpentanoic acid (0.14 mmol) was then added to the auristatin E solution. Reaction was performed overnight at room temperature. The product was purified and separated using preparative thin layer chromatography using 5% methanol in ethyl acetate as developing solvent.

Step3. Synthesis of hydrazone intermediate: Benzoylpentanoic ester of auristatin E (1eq) and maleimidocaproyl hydrazide (5eq) was added in anhydrous methanol with 0.01% acetic acid. The reaction mixture was stirred overnight at room temperature. The reaction mixture was added into DMSO and only methanol was evaporated under reduced pressure. Finally, the product was purified using semi-preparative column in RP HPLC using gradient of triethyl ammonium acetate buffer pH 7 and acetonitrile.

Step 4. Synthesis of peptide-drug conjugate: Peptide with a free thiol group and hydrazone intermediate were dissolved separately in tert-butyl alcohol and 50 mM ammonium bicarbonate buffer pH 7.4 (1:3). The pH of both solutions were adjusted to 7.4. Peptide was added to the hydrazone complex solution dropwise and reaction mixture was stirred for 10 minutes. Reaction mixture was then directly purified through HPLC using gradient of triethyl ammonium acetate (TEAA) buffer at pH=7 and acetonitrile and finally the product was lyophilized (see supplementary file for structures).

### *In vitro* cytotoxicity of peptide-drug conjugate

The cytotoxicity study of MMAE, peptide, peptide drug conjugate and Cetuximab was performed using A431, MDA MB 468 cells and HEK 293. Cells were cultured to 80% confluency in T75 culture flasks using DMEM as growth medium, respectively. The cells were seeded onto 96 well plates and grown for 24 h to reach 50% confluency. This was followed by treatment with various concentrations of drug MMAE, peptide drug conjugate, peptide and Cetuximab ranging from 0.00001 to 10000 nM for 72 hours at 37 °C.

At the end of the incubation period, the cells were fixed using 10% trichloroacetic acid, followed by washing with distilled water and drying. The cellular proteins were stained using 50 μL of 0.4% Sulforhodamine B (SRB) in 1% acetic acid. Unbound SRB was washed with 1% acetic acid and the plates were dried overnight. The cell bound SRB was then solubilized using 200 μL of 10 mM unbuffered tris base solution. SRB absorbance was measured at 560 nm wavelength using a plate reader. The percent viabilities of cells were plotted as a function of log concentration and data was analyzed in GraphPad Prism Version 5.0 software (GraphPad Software Inc, CA, USA) using nonlinear-regression curve fit (variable slope four parameter equation).

## Supplementary information

A Rational Approach for Creating Peptides Mimicking Antibody Binding
