# Supplementary material for: A Rational Approach for Creating Peptides Mimicking Antibody Binding
Source: Sci Rep. 2019 Jan 30;9:997. doi: 10.1038/s41598-018-37201-6 (PMC6353898; doi:10.1038/s41598-018-37201-6)
Supplement: Supplementary file 1 — A Rational Approach for Creating Peptides Mimicking Antibody Binding [file 41598_2018_37201_MOESM1_ESM.docx]

**Supplementary Information**

**A Rational Approach for Creating Peptides Mimicking Antibody Binding**

Sameer Sachdeva^1,3^, Hyun Joo^2^, Jerry Tsai^2^, Bhaskara Jasti^1^, Xiaoling Li^1, *^

^1^Department of Pharmaceutics and Medicinal Chemistry, University of the Pacific, Stockton, CA 95211, USA

^2^Department of Chemistry, University of the Pacific, Stockton, CA 95211, USA

^3^Current Affiliation-Amneal Pharmaceuticals, Piscataway, NJ 08854, USA

^*^Corresponding author

| Supplementary Table 1. Peptide sequence with docking results. | | | | |
| --- | --- | --- | --- | --- |
| No. | Peptide | Energy  (kcal/mol) | Total Interactions | Preserved  Interactions |
| 1. 3. | WSGENGPGYYDYEA | -35.80 | 13 | 7 |
| 1. 4. | WSGENGPGYWDYEA | -24.54 | 13 | 6 |
|  | WSGENGPGYLDYEA | -33.82 | 13 | 5 |
|  | WSGENGPGYIDYEA | -28.95 | 13 | 7 |
|  | WSGENGPGYVDYEA | -28.42 | 13 | 3 |
|  | WSGENGPGYFDYEA | -32.47 | 13 | 4 |
|  | WSGENGPGWYDYEA | -35.91 | 13 | 4 |
|  | WSGENGPGLYDYEA | -28.28 | 14 | 4 |
|  | WSGENGPGIYDYEA | -26.00 | 14 | 3 |
|  | WSGENGPGVYDYEA | -28.39 | 13 | 6 |
|  | WSGENGPGFYDYEA | -40.43 | 17 | 8 |
|  | WSGENGPGTYYDYEA | -31.28 | 14 | 6 |
|  | WSGENGPGTYWDYEA | -28.78 | 12 | 4 |
|  | WSGENGPGTYLDYEA | -35.62 | 14 | 5 |
|  | WSGENGPGTYIDYEA | -31.25 | 10 | 2 |
|  | WSGENGPGTYVDYEA | -34.33 | 13 | 2 |
|  | WSGENGPGTYFDYEA | -37.13 | 13 | 6 |
|  | WSGENGPGTWYDYEA | -37.94 | 13 | 7 |
|  | WSGENGPGTLYDYEA | -31.89 | 14 | 7 |
|  | WSGENGPGTIYDYEA | -29.32 | 12 | 4 |
| 21. | WSGENGPGTVYDYEA | -36.50 | 14 | 6 |
| 22 | WSGENGPGTFYDYEA | -35.34 | 14 | 6 |
| 23 | AEYDYFGPGNEGSW | -30.92 | 10 | 3 |
| 24 | AEYDFYGPGNEGSW | -40.31 | 13 | 4 |
| 25  Control | SGEWAYDGYEPNFG | -23.69 | 7 | 2 |

Supplementary Table 2. Knobs with frequency of binding for ISI socket.

| Knobs | W | P | G | Y | F | V | I | L | A | E | K | R | Q | D | S | T | N | M | H | C |
| --- | --- | --- | --- | --- | --- | --- | --- | --- | --- | --- | --- | --- | --- | --- | --- | --- | --- | --- | --- | --- |
| Frequency | 0 | 1 | 1 | 7 | 0 | 3 | 2 | 1 | 2 | 2 | 1 | 0 | 4 | 0 | 1 | 3 | 4 | 2 | 2 | 0 |


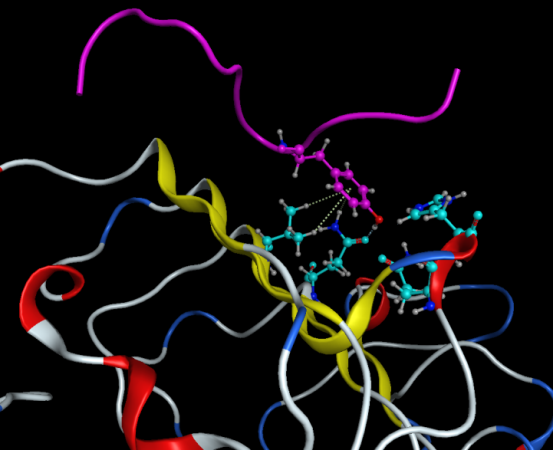

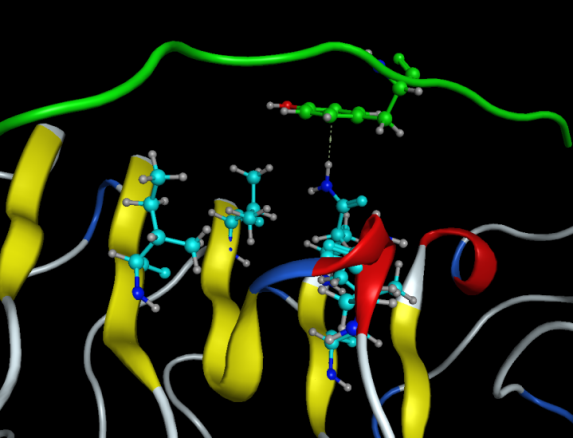


(a) (b)


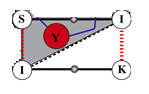


(c)

Supplementary Figure 1. Interactions of first tyrosine residue in Pep11 showing 4 interactions (a) and Pep22 showing 1 interaction with EGFR (b). Tyrosine (Y) knob in the ISI socket (c).


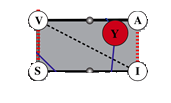


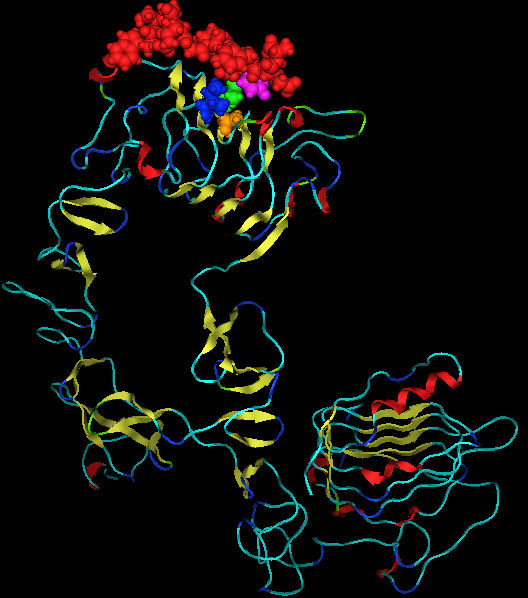

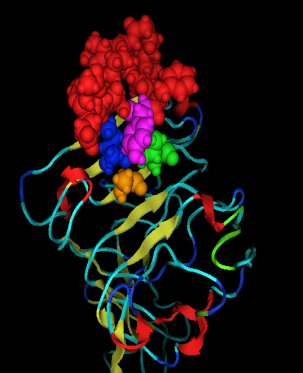


(a) (b)

Supplementary Figure 2. Docking image of Pep11 displaying knobs and sockets. Pep11 is shown after docking it to the same site as Cetuximab to the EGFR. Tyrosine residue (pink) from Pep11 is representing a knob which is fitting into the socket formed by alanine (orange), valine (green) and isoleucine (blue) on EGFR epitope (a). Sockets alanine, valine and isoleucine on EGFR epitope are shown in white and knob tyrosine in red from peptide design (b).

**Synthesis of Peptide Drug Conjugate**


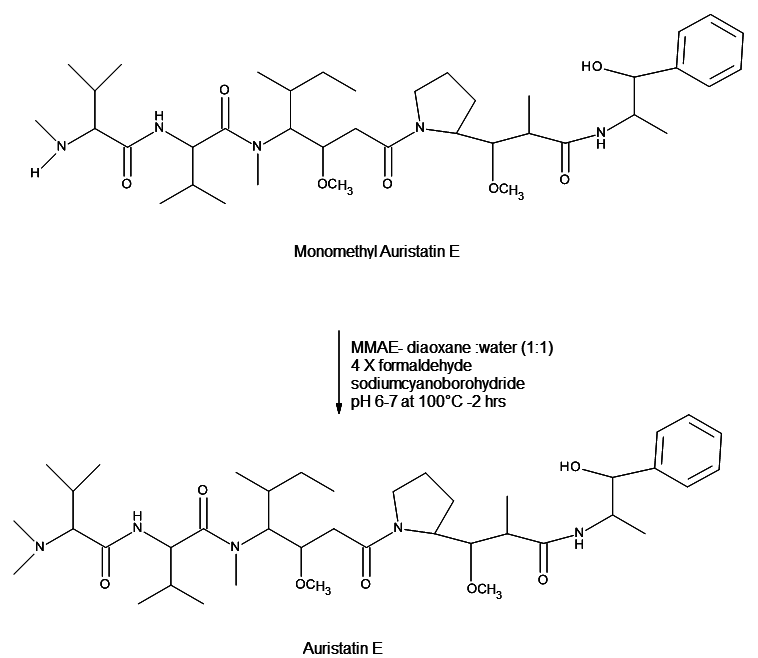


Supplementary Figure 3. Synthesis of Auristatin E


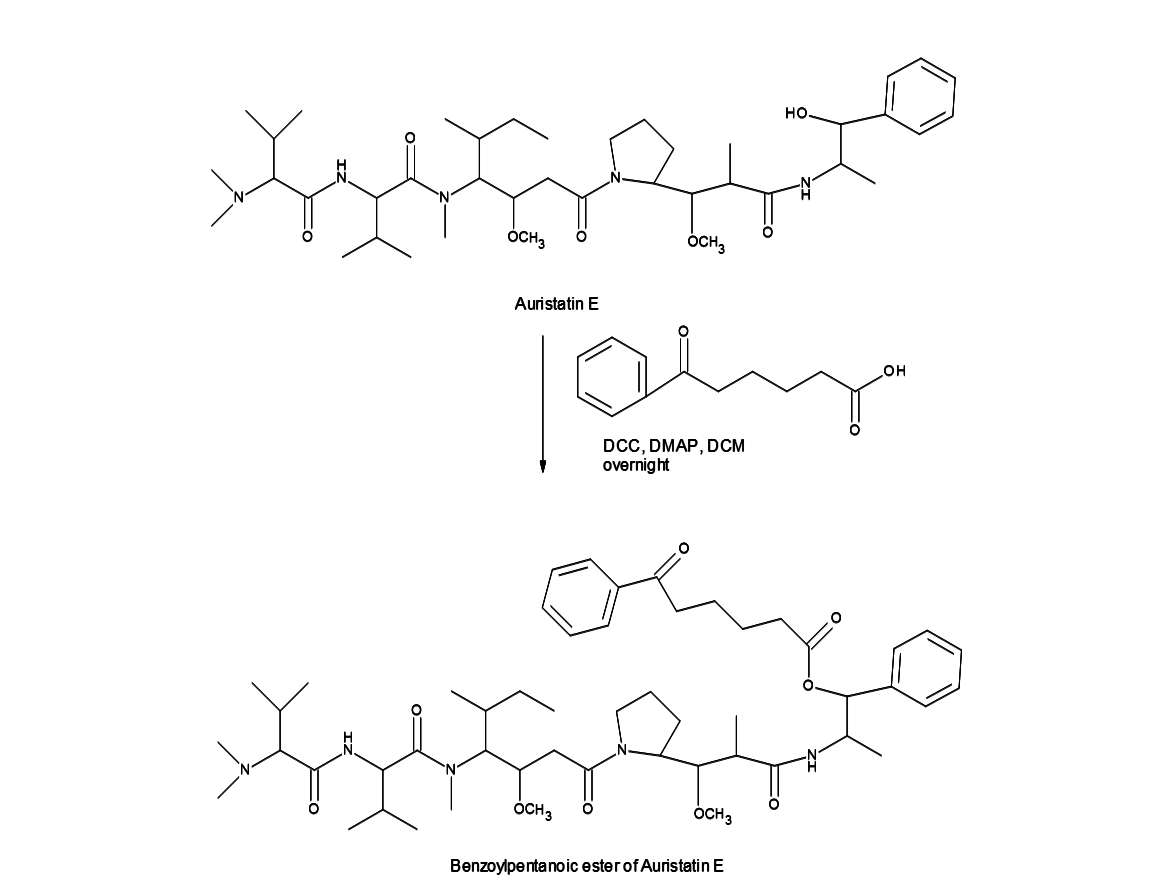


Supplementary Figure 4. Synthesis of 5-benzoylpentanoic ester of auristatin E (BPA)


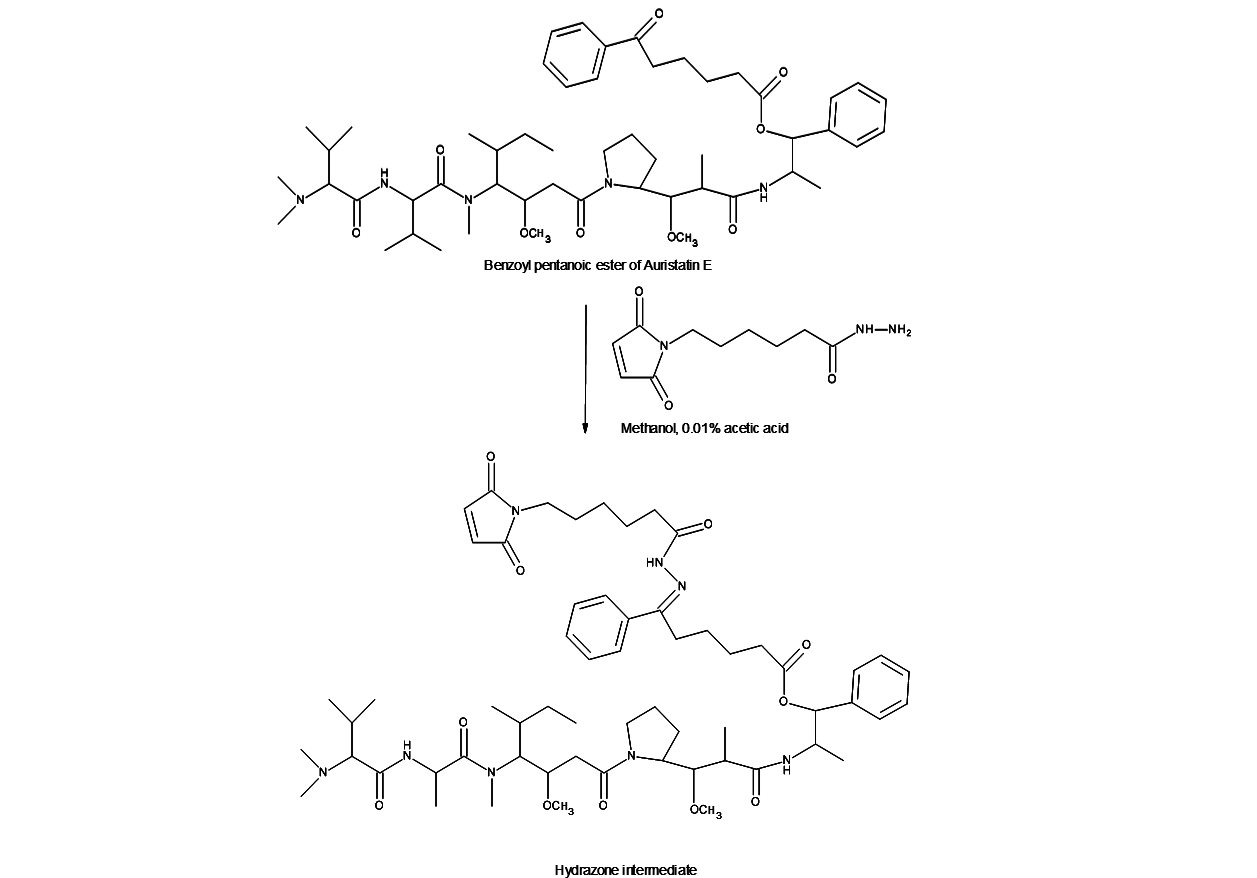


Supplementary Figure 5. Synthesis of hydrazone intermediate


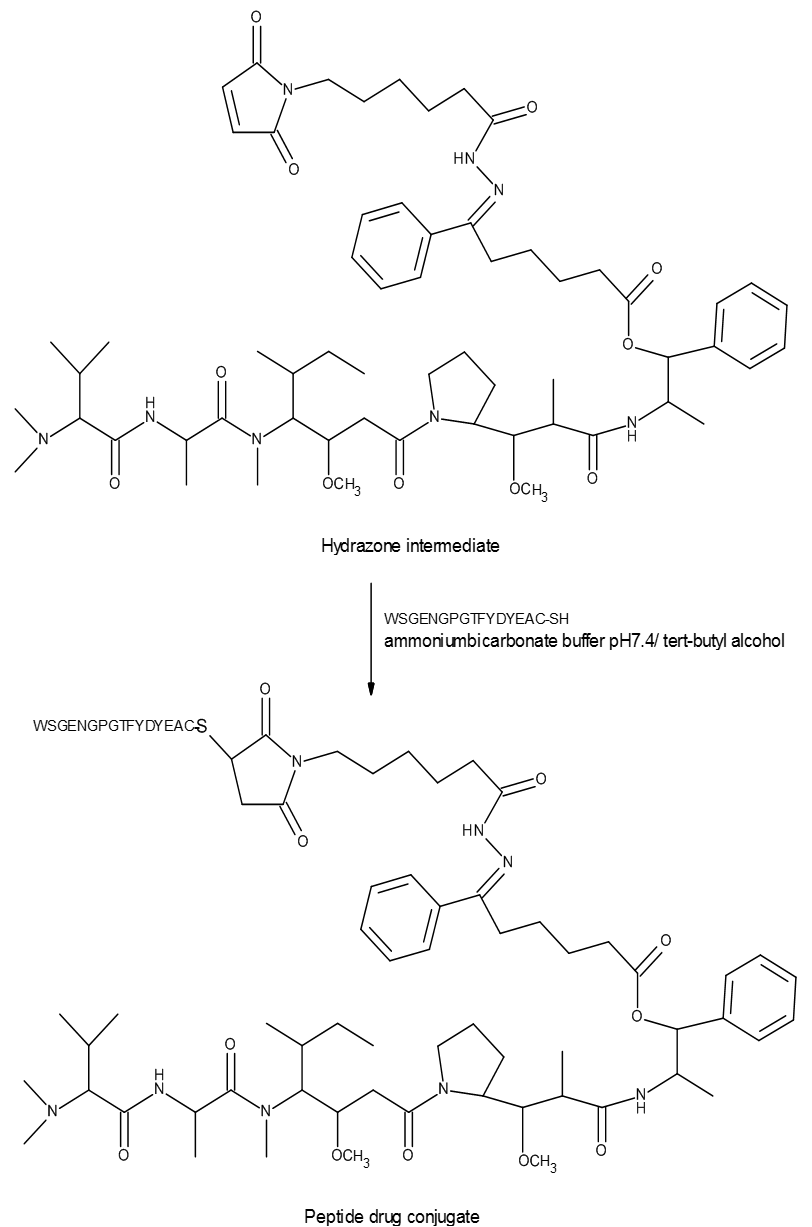


Supplementary Figure 6. Synthesis of peptide drug conjugate
